# Determinants of organised sports participation patterns during the transition from childhood to adolescence in Germany: results of a nationwide cohort study

**DOI:** 10.1186/s12889-016-3615-7

**Published:** 2016-09-06

**Authors:** Kristin Manz, Susanne Krug, Anja Schienkiewitz, Jonas David Finger

**Affiliations:** Department of Epidemiology and Health Monitoring, Robert Koch-Institute, PO Box 650261, 13302 Berlin, Germany

**Keywords:** Organised sports, Dropout, Predictors, Children, Adolescents, Germany

## Abstract

**Background:**

Organised sports (OS) participation is an important health behaviour but it seems to decline from childhood to adolescence. The aim of this study was to investigate OS participation patterns from childhood to adolescence and potential determinants for those patterns.

**Methods:**

Data from the German Health Interview and Examination Survey for Children and Adolescents (KiGGS) cohort study with a 6 year follow-up period were used (KiGGS0: 2003-06, KiGGS1: 2009-12). Participants aged 6–10 years at KiGGS0, who were aged 12–16 at KiGGS1, were included (*n* = 3790). The outcome variable was ‘OS participation’ between KiGGS0 and KiGGS1 with the categories ‘maintenance’ (reference), ‘dropout’, ‘commencement’ and ‘nonparticipation’. Relative risk ratios (RRRs) were calculated using multinomial logistic regression to identify potential predictors for OS patterns. Socio-demographic, family-related, health-related, behavioural and environmental factors were considered as independent variables.

**Results:**

48.5 % maintained OS, 20.5 % dropped out, 12.3 % commenced OS between KiGGS0 and KiGGS1 and 18.7 % did not participate at both times. The RRRs for dropout rather than maintenance were 0.6 (95 % Cl 0.5–0.7) for boys versus girls, 1.5 (1.3–1.9) for the age group 8–10 versus 6–7 years, 0.7 (0.5–0.9) for high versus intermediate parental education, 1.4 (1.1–1.8) for low versus middle household income, 1.4 (1.0–1.8) for below-average versus average motor fitness. The RRRs for commencement rather than maintenance were 0.6 (0.5–0.8) for boys versus girls, 0.6 (0.5–0.8) for the age group 8–10 versus 6–7 years, 1.5 (1.1–2.1) for low versus intermediate parental education, 1.5 (1.1–2.0) for low versus middle household income, 0.7 (0.5–1.0) for no single-parent versus single parent family, 1.8 (1.3–2.5) for below-average and 0.6 (0.4–0.8) for above-average versus average motor fitness, and 1.4 (1.1–1.9) for high versus middle screen-based media use. The RRRs for abstinence rather than maintenance were 0.6 (0.4–0.7) for boys versus girls, 1.5 (1.1–2.0) for low versus intermediate parental education, 2.2 (1.7–2.8) for low and 0.6 (0.5–0.8) for high versus middle household income, 1.6 (1.2–2.1) for psychopathological problems versus no problems, 1.7 (1.3–2.2) for below-average and 0.4 (0.3–0.6) for above-average versus average motor fitness, and 1.6 (1.0–2.6) for rural versus metropolitan residential area.

**Conclusions:**

OS participation rates among all children living in Germany need to be improved. More tailored offerings are needed which consider the preferences and interests of adolescents as well as a cooperation between public health actors to reduce barriers to OS.

**Electronic supplementary material:**

The online version of this article (doi:10.1186/s12889-016-3615-7) contains supplementary material, which is available to authorized users.

## Background

Regular physical activity (PA) during childhood and adolescence is associated with numerous short- and long-term health benefits [1, 2]. Studies indicate that a dose-response relationship exists: the larger the amount and the higher the intensity level of PA, the greater the health benefits [2]. Organised sports (OS) such as organised team sports seem to have greater health benefits compared to non-organised PA because their PA intensity level is usually higher than that of non-organised PA [3]. Furthermore, the total amount of leisure-time PA usually is greater among OS participants compared to nonparticipants [3, 4]. It seems that OS especially have a positive effect on mental health because of the various social interactions that are particularly associated with them [5, 6]. Thus, there is a general consensus that OS should be an integral part of children’s and adolescents’ daily life. It is well documented, however, that OS participation declines during adolescence [7–9]. Recent population based data for German children and adolescents demonstrate that the prevalence was higher in the age group 7 to 10 years with 69.2 % than in the age groups 11 to 13 and 14 to 17 years with 61.2 and 55.7 % [10]. Cohort data analyses are needed to investigate changes in OS participation during the transition from childhood to adolescence, as well as their determinants, to identify target groups for health promotion interventions. The hierarchal leisure constraint model [11] and the socio ecological model of sport attrition [12] identify biological (sex, body mass index [BMI]), intra- and interpersonal (attitude, anxiety, fun, social support, pressure) as well as structural factors (environment, socioeconomic status [SES], costs) that could inhibit or prevent leisure-time PA and sport attrition. Authors of a review study on correlates of youth sport attrition concluded that most studies reviewed examined intra- and interpersonal correlates whereas studies on biological and environmental correlates were underrepresented [12].

This study is aimed at investigating patterns of OS participation (maintenance, dropout, commencement and nonparticipation) during the transition from childhood to adolescence. Furthermore, the role of influencing factors for OS participation patterns is investigated, with a focus on socio-demographic, family-related, health-related, behavioural, and environmental factors.

## Methods

### Study design and participants

Data from the German Health Interview and Examination Survey for Children and Adolescents (Kinder- und Jugendgesundheitssurvey, KiGGS) baseline survey (KiGGS0) and the 6 years later conducted first follow-up survey (KiGGS1) were analysed. KiGGS is a nationwide cohort study based on a cluster-randomized population survey design, with the aim of obtaining comprehensive, nationally representative information on the health status of children and adolescents living in Germany. The sample was drawn with a two-stage sampling strategy; probability selection of 167 sample points (clusters) and random selection of address data from local population registries within the clusters [13]. The KiGGS study design is described in detail elsewhere [13]. In KiGGS0, 17,641 children and adolescents aged between 0 and 17 years underwent physical examinations, interviews and tests between May 2003 and May 2006. The parents of the participants also completed a self-administered questionnaire. The response rate of KiGGS0 was 66.6 %. The study protocol was approved by the ethics committee of the Charité - University Medicine Berlin and the Federal Commissioner for Data Protection and Freedom of Information. In KiGGS0 and KiGGS1 participants were informed about the study goals, data protection protocols and the interview and examination processes. All participants gave their informed consent and one parent signed an informed written consent.

**Fig. 1:**
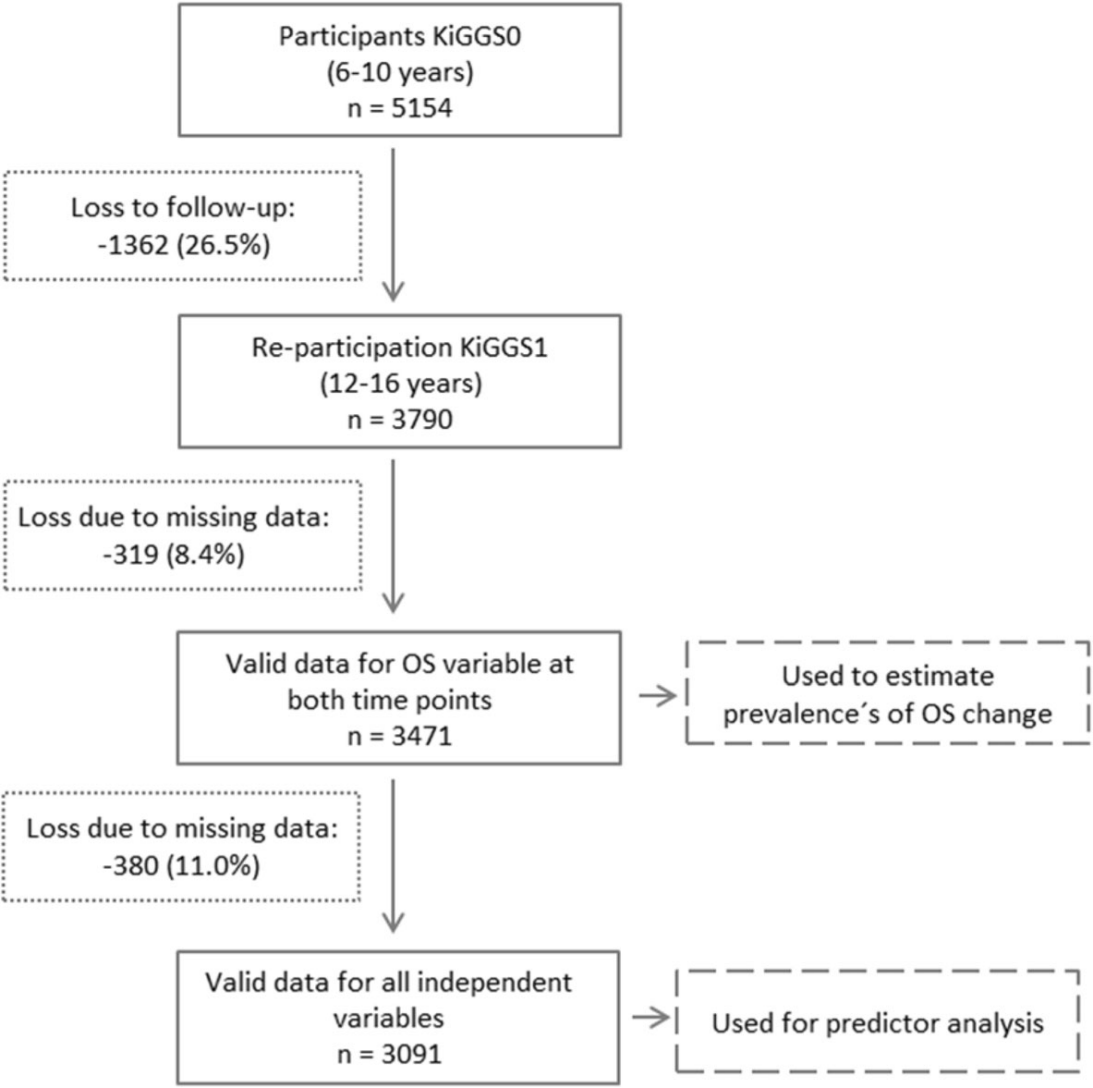
Flow diagram of participants

KiGGS1 was carried out as a computer-assisted telephone interview (CATI) survey 6 years after KiGGS0, between June 2009 and June 2012 [13, 14]. For KiGGS1, all KiGGS0 participants were re-invited. The re-participation rate among persons aged between 7 and 17 years in KiGGS1 was 72.9 % (*n* = 7913) [13, 14]. This particular study included 3790 participants aged between 6 and 10 years at KiGGS0 and then between 12 and 16 years at KiGGS1 (Fig. 1). The loss to follow-up for this particular age group was 26.5 %. Characteristics of study participants and those who were lost to follow-up are compared in Additional file 1. The final study sample comprised of 3471 (91.6 %) participants for whom complete data on OS participation was available for both KiGGS0 and KiGGS1. The predictor analysis was conducted as a complete-case analysis to allow comparison between the models. Hence, participants with missing data for one or more predictor variables were excluded (n_included_ = 3091; 11.0 % excluded; for further information see item nonresponse analysis in the Additional file 2).

### Measurements and quantitative variables

#### Outcome measure

In KiGGS0, OS participation was assessed via a self-administered questionnaire completed by a parent. The parent was asked how often her/his child participates in sports club activities with the response categories ‘almost every day’, ‘3–5 times per week’, ‘1–2 times per week’, ‘less than once per week’ and ‘never’. A dichotomous variable was constructed: ‘no organised sports participation’ if ‘never’ was chosen and ‘organised sports participation’ if one of the other categories were chosen.

In KiGGS1, 12–16 year old adolescents were first asked via telephone interview whether they did sports activities which were not part of the physical education during school and next asked whether their sports activities took place in a sports club, outside of a sports club or both, in and outside of a sports club. In the question wordings no further specification of sports was provided. In the German context the term ‘sport’ has a broad meaning including sports and exercises activities. ‘No organised sports participation’ was defined if the participants did not do any sports or if their sports activity took place outside of a sports club. ‘Organised sports participants’ was defined if the participants indicated to do sports ‘in a sports club’ or ‘in and outside of a sports club’.

An ‘OS participation’ variable was generated combining the information from KiGGS0 and KiGGS1 into four categories: ‘maintenance’, if the child participated in OS at KiGGS0 and KiGGS1; ‘dropout’, if the child participated in OS at KiGGS0 but not at KiGGS1; ‘commencement’, if the child did not participate in OS at KiGGS0 but did at KiGGS1; ‘nonparticipation’, if the child did not participate in OS at both KiGGS0 and KiGGS1.

#### Predictor variables

Potential predictor variables for OS participation were selected based on evidence in the literature [11, 12]. All information used for constructing the predictor variables was collected at KiGGS0 either with self-administered questionnaires filled in by one parent or during physical examination of the children.

#### Socio-demographic factors

‘Parental education level’ was constructed based on the highest school-completion certificate and the highest vocational-training certificate achieved by either the mother or father of the participant. A categorical parental education level variable (low, intermediate, high) was constructed, the methodology for which is described in detail elsewhere [15].

‘Household-equivalent income’ was calculated based on the households’ approximate monthly net income and the number of individuals living permanently in the household [15]. A categorical household income level variable was constructed by calculating tertiles of the household-equivalent income variable (low, middle, high).

‘Migrant background’ was defined if the participant had immigrated to Germany and if at least one parent was not born in Germany or if both parents had immigrated to Germany or both parents did not hold German citizenship [16].

#### Family-related factors

Family form was defined as ‘single-parent family’ if the participant lived together with a single mother or a single father, and no new partner of the parent lived in the household.

#### Health-related factors

Body weight and height were measured using standardized methodology. A ‘weight status’ variable was constructed by calculating participants’ BMI and classifying the participants into ‘overweight or obesity’ or ‘no overweight or obesity’ categories according to the national BMI reference values of Kromeyer-Hauschild [17].

For the parental evaluation of the participants’ health status, one parent was asked, ‘How would you describe the general health status of your child?’, with the response categories ‘very good’, ‘good’, ‘fair’, ‘poor’ and ‘very poor’. A dichotomous ‘general health status’ variable was constructed with the categories ‘very good’ and ‘not very good’, with ‘not very good’ comprising of the responses good, fair, poor and very poor.

‘Special health care needs’ of the participants were assessed by a German version of the ‘Child with Special Health Care Needs’ screener (CSHCN) [18, 19]. The CSHCN identifies children who suffer from long-term health problems that require health services or cause functional limitations.

The ‘Strengths and Difficulties Questionnaire’ was used to assess ‘psychopathological problems’ among the participants, based on information on emotional, behavioural, peer and hyperactivity problems as well as prosocial behaviour [20, 21]. Three outcome categories (normal, borderline, abnormal) were summarized into a dichotomous variable with the categories ‘psychopathological problems’ (borderline and abnormal) and ‘no psychopathological problems’ (normal).

‘Motor fitness’ of the participants was measured with the motoric test ‘jumping sideways’ which measures total body coordination under time pressure, as well as speed and muscular endurance of the lower extremities [22]. A categorical variable based on the age- und sex-specific percentiles was constructed: < 20th percentile, ‘below-average fitness’; ≥ 20th - ≤ 80th percentile, ‘average fitness’; > 80th percentile, ‘above-average fitness’.

#### Behavioural factor

One parent was asked to indicate the average duration that their child spent watching TV or videos and playing computer games on weekdays and on weekends. A ‘screen-based media use’ index (average duration per day) was calculated and a categorical variable was constructed by calculating tertiles (low, middle, high).

#### Environmental factor

A ‘residential area size’ variable was constructed with four categories: < 5000 inhabitants, ‘rural area’; 5000 - < 20,000 inhabitants, ‘small-sized city’; 20,000 - < 100,000 inhabitants, ‘medium-sized city’; ≥ 100,000 inhabitants, ‘metropolitan city’.

### Statistical analyses

All analyses were performed with the survey design procedures of Stata/SE 14.0 to adjust for the cluster design. Analyses were standardized to the age structure of the above mentioned age group of the German population on December 31, 2004. To compensate for different re-participation rates study-specific weighting factors were calculated and used to describe the distribution of the outcome indicator, OS participation, in the study sample. A ‘lasagne plot’ [23] was created to illustrate the distribution of OS participation between KiGGS0 and KiGGS1. Multinomial logistic regression was used to analyse the influence of a predictor variable on all categories of the dependent variable (maintenance, dropout, commencement and nonparticipation) at the same time by selecting the ‘maintenance’ category of the dependent variable as the reference category. As a first step, binary analyses were performed to evaluate the statistical significance of potential predictors for OS participation; these were subsequently included in the multivariate models if they were significantly associated (*p* < .2) with the outcome variable for at least one group comparison (e.g., maintenance versus dropout). As a next step, all preselected predictors of the respective outcomes were successively included into multinomial regression models: Model 1, socio-demographic factors (sex, age, parental educational level, household income, and migrant background); Model 2, the family-related factor ‘family form’ was added; Model 3, health-related factors (weight status, general health status, special health care needs, psychopathological problems, and motor fitness) were added; Model 4, the behavioural factor ‘screen-based media use’ was added; Model 5 the environmental factor ‘residential area size’ was added. The Relative Risk Ratios (RRRs) and associated 95 % confidence intervals (CIs) for each successive model are reported.

Because of relatively small sample sizes in subgroups, the prediction analysis was performed without stratifying by sex. In the final model (Model 5), interaction terms (sex*predictor variables) were included to test whether sex is an effect modifier for the associations observed.

## Results

Descriptive characteristics for the total study sample and for girls and boys separately are shown in Table 1. Girls comprised of 49.1 % of the study sample, the mean age was 8.5 years and 9.3 % had a migrant background.

**Table 1:** Descriptive characteristics of the study sample at KiGGS0

Characteristics	Girls (*n* = 1705)	Boys (*n* = 1766)	Total (*n* = 3471)
Age in years (mean)	8.4	8.5	8.5
Parental education (%)			
Low	12.3	11.2	11.8
Intermediate	55.7	55.3	55.5
High	32.0	33.4	32.8
Household income (%)			
Low	31.5	32.1	31.8
Middle	37.4	37.9	37.6
High	31.1	30.0	30.5
Migrant background (% yes)	8.8	9.8	9.3
Family form (% single parent)	9.3	8.8	9.1
Overweight/obesity (% yes)	12.4	13.3	12.8
General health status (% not very good) *	54.6	60.1	57.4
Special health care needs (% yes) *	11.6	20.1	15.9
Psychopathological problems (% yes) *	17.9	10.1	14.1
Motor fitness (%)			
Below-average	16.2	17.3	16.8
Average	65.6	63.6	64.6
Above-average	18.3	19.0	18.7
Screen-based media use (%) *			
Low	42.9	31.7	37.2
Middle	34.3	35.2	34.8
High	22.8	33.1	28.0
Residential area size (%)			
Rural	22.4	21.8	22.1
Small-sized city	27.9	29.0	28.4
Medium-sized city	29.9	28.5	29.2
Metropolitan city	19.9	20.7	20.3

*boys and girls significantly different, *p* < .05 (Chi-square-test with Rao-Scott correction)

### OS participation from childhood to adolescence

**Fig. 2:**
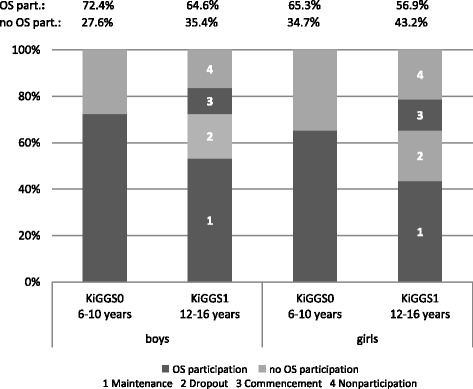
Distribution of OS participation according to survey wave stratified by sex

The weighted prevalence proportions across OS groups indicate that almost half of the participants (48.5 %) maintained their OS between KiGGS0 and KiGGS1, 20.5 % dropped their OS, 12.3 % commenced OS and 18.7 % did not participate at both KiGGS0 and KiGGS1. Boys were more likely to maintain their OS compared to girls (*p* < .001) and were less likely to not participate (*p* = .001). OS participation changes from KiGGS0 to KiGGS1 stratified by sex are illustrated in Fig. 2.

The distributions of OS participation according to socio-demographic, family-related, health-related, behavioural and environmental variables are presented in Table 2.

**Table 2:** Weighted prevalence proportions of OS participation by socio-demographic, family-related, health-related, behavioural, and environmental variables

	Number	Missing	Maintenance % (95 % Cl)	Dropout % (95 % Cl)	Commencement % (95 % Cl)	Nonparticipation % (95 % Cl)
Total sample	3471		48.5 (46.2–50.8)	20.5 (18.7–22.4)	12.3 (10.8–14.0)	18.7 (16.8–20.8)
Sex		0				
Boys	1766		53.3 (50.1–56.5)	19.1 (16.7–21.7)	11.3 (9.4–13.5)	16.3 (14.0–18.9)
Girls	1705		43.5 (40.3–46.7)	21.9 (19.5–24.5)	13.4 (11.4–15.7)	21.3 (18.4–24.5)
Age group		0				
8–10 years	2043		48.2 (45.0–51.3)	23.2 (20.8–25.7)	9.7 (8.2–11.5)	19.0 (16.5–21.7)
6–7 years	1428		48.9 (45.6–52.2)	16.7 (14.1–19.7)	15.9 (13.4–18.8)	18.5 (15.5–21.8)
Parental education		8				
Low	407		35.1 (30.0–40.5)	18.2 (14.2–23.0)	15.9 (12.3–20.2)	30.9 (26.5–35.8)
Intermediate	1922		50.0 (47.3–52.6)	23.4 (21.3–25.6)	11.1 (9.6–12.9)	15.6 (13.6–17.7)
High	1134		63.1 (59.5–66.6)	17.6 (15.1–20.5)	10.1 (8.2–12.4)	9.2 (7.6–11.0)
Household income		19				
Low	1099		35.2 (31.5–39.2)	20.7 (17.7–24.1)	14.4 (11.7–17.5)	29.7 (26.2–33.5)
Middle	1299		54.6 (50.9–58.1)	19.1 (16.6–21.9)	12.4 (10.2–15.1)	13.9 (11.9–16.2)
High	1054		61.7 (58.0–65.3)	22.3 (19.3–25.7)	8.9 (7.0–11.2)	7.0 (5.4–9.1)
Migrant background		14				
Yes	321		35.0 (28.3–42.4)	20.9 (15.5–27.6)	12.8 (8.3–19.2)	31.3 (25.3–38.1)
No	3136		51.0 (48.5–53.4)	20.4 (18.5–22.3)	12.2 (10.6–14.0)	16.5 (14.5–18.6)
Family form		0				
No single-parent	3156		49.6 (47.1–52.2)	20.4 (18.5–22.4)	12.3 (10.6–14.1)	17.7 (15.8–19.8)
Single-parent	315		39.5 (32.8–46.6)	21.0 (16.0–27.1)	12.9 (8.9–18.4)	26.7 (19.9–34.8)
Overweight/obesity		12				
Yes	444		38.7 (33.2–44.5)	23.5 (18.5–29.3)	13.3 (9.2–18.9)	24.5 (18.6–31.7)
No	3015		50.0 (47.6–52.3)	20.0 (18.1–22.1)	12.2 (10.6–14.0)	17.8 (15.9–20.0)
General health status		12				
Not very good	1986		46.1 (43.0–49.2)	21.2 (19.2–23.5)	13.0 (11.1–15.2)	19.7 (17.2–22.6)
Very good	1473		52.3 (48.9–55.7)	19.7 (17.3–22.3)	11.5 (9.5–13.9)	16.5 (13.7–19.7)
Special health care needs		173				
Yes	523		49.8 (47.1–52.5)	20.8 (18.8–22.9)	12.0 (10.4–13.9)	17.4 (15.4–19.7)
No	2775		44.5 (39.2–49.9)	22.1 (18.5–26.3)	14.1 (10.4–19.0)	19.3 (15.0–24.5)
Psychopathological problems		11				
Yes	2972		50.8 (48.2–53.3)	20.3 (18.3–22.5)	11.8 (10.2–13.6)	17.2 (15.1–19.4)
No	488		38.0 (32.8–43.6)	20.9 (16.7–25.8)	15.1 (10.8–20.8)	26.0 (21.2–31.6)
Motor fitness		51				
Below-average	573		32.7 (28.2–37.6)	20.0 (16.4–24.3)	16.6 (12.9–21.1)	30.7 (25.4–36.4)
Average	2209		48.3 (45.4–51.2)	21.4 (19.0–23.9)	11.8 (10.0–13.9)	18.5 (16.1–21.1)
Above-average	638		63.1 (58.0–68.0)	18.4 (15.4–21.8)	9.9 (7.1–13.7)	8.6 (5.9–12.3)
Screen-based media use		136				
Low	1242		54.8 (51.1–58.4)	19.8 (16.9–23.1)	11.0 (8.9–13.5)	14.4 (12.0–17.1)
Middle	1159		51.0 (46.9–55.1)	19.5 (16.8–22.6)	12.4 (10.0–15.2)	17.1 (14.2–20.5)
High	934		41.1 (37.3–45.0)	21.4 (18.3–24.8)	14.0 (11.1–17.4)	23.6 (19.8–27.9)
Residential area size		0				
Rural area	766		48.2 (40.7–55.8)	19.6 (15.0–25.3)	13.9 (10.5–18.3)	18.2 (13.5–24.2)
Small-sized city	987		51.0 (47.3–54.7)	20.6 (17.2–24.4)	10.3 (7.6–13.9)	18.1 (15.2–21.4)
Medium-sized city	1013		50.6 (47.0–54.2)	22.3 (19.7–25.2)	11.5 (9.2–14.4)	15.5 (12.8–18.8)
Metropolitan city	705		43.5 (39.3–47.8)	18.6 (15.3–22.6)	14.3 (11.1–18.2)	23.6 (19.5–28.2)

### Predictor analysis

The binary analyses revealed that all potential predictor variables selected were associated with the OS outcome variable for one or more group comparisons (multinomial regression) and were thus considered during the multivariate analysis. During the stepwise multinomial logistic regression, the associations between the predictor and outcome variables mostly remained unchanged when further predictor variables were added. The only exceptions were ‘weight status’, which was a predictor for nonparticipation in Model 3, but not in Models 4 and 5, and ‘family form’, which was not a predictor for commencement in Models 2–4, but was a predictor in Model 5. The results of the final model with all predictors included are presented in Table 3.

**Table 3:** Results of the final multinomial logistic regression model for the prediction of OS participation (reference group: maintenance)

Model	Variable	Dropout RRR (95 % Cl)	Commencement RRR (95 % Cl)	Nonparticipation RRR (95 % Cl)
1	Sex			
	Boys	0.6 (0.5–0.7)*	0.6 (0.5–0.8)*	0.6 (0.4–0.7)*
	Girls	1.0	1.0	1.0
	Age group			
	8–10 years	1.5 (1.3–1.9)*	0.6 (0.5–0.8)*	1.0 (0.8–1.2)
	6–7 years	1.0	1.0	1.0
	Parental education			
	Low	0.9 (0.7–1.3)	1.5 (1.1–2.1)*	1.5 (1.1–2.0)*
	Intermediate	1.0	1.0	1.0
	High	0.7 (0.5–0.9)*	1.1 (0.8–1.5)	1.1 (0.9–1.4)
	Household income			
	Low	1.4 (1.1–1.8)*	1.5 (1.1–2.0)*	2.2 (1.7–2.8)*
	Middle	1.0	1.0	1.0
	High	1.1 (0.9–1.4)	0.8 (0.6–1.1)	0.6 (0.5–0.8)*
	Migrant background			
	Yes	1.3 (0.9–1.9)	1.2 (0.7–1.9)	1.4 (0.9–2.1)
	No	1.0	1.0	1.0
2	Family form			
	No single-parent	0.8 (0.6–1.2)	0.7 (0.5–1.0)*	0.7 (0.5–1.0)
	Single-parent	1.0	1.0	1.0
3	Overweight/obesity			
	Yes	1.1 (0.8–1.5)	1.0 (0.7–1.5)	1.3 (1.0–1.8)
	No	1.0	1.0	1.0
	General health status			
	Not very good	1.2 (1.0–1.4)	1.2 (0.9–1.5)	1.2 (1.0–1.4)
	Very good	1.0	1.0	1.0
	Special health care needs			
	Yes	1.2 (1.0–1.6)	1.2 (0.8–1.7)	0.9 (0.7–1.3)
	No	1.0	1.0	1.0
	Psychopathological problems			
	Yes	1.2 (0.9–1.6)	0.9 (0.6–1.4)	1.6 (1.2–2.1)*
	No	1.0	1.0	1.0
	Motor fitness			
	Below-average	1.4 (1.0–1.8)*	1.8 (1.3–2.5)*	1.7 (1.3–2.2)*
	Average	1.0	1.0	1.0
	Above-average	0.7 (0.5–0.9)*	0.6 (0.4–0.8)*	0.4 (0.3–0.6)*
4	Screen-based media use			
	Low	0.9 (0.7–1.2)	1.0 (0.7–1.3)	0.9 (0.7–1.2)
	Middle	1.0	1.0	1.0
	High	1.2 (0.9–1.5)	1.4 (1.1–1.9)*	1.3 (1.0–1.7)
5	Residential area size			
	Rural area	1.0 (0.7–1.4)	1.4 (0.9–2.2)	1.6 (1.0–2.6)*
	Small-sized city	0.9 (0.7–1.2)	0.8 (0.6–1.2)	1.0 (0.7–1.5)
	Medium-sized city	1.0 (0.7–1.2)	0.9 (0.6–1.3)	1.0 (0.7–1.4)
	Metropolitan city	1.0	1.0	1.0

*p < .05; *n* = 3091

#### Socio-demographic factors

Boys were less likely to be in the dropout, commencement and nonparticipation OS groups rather than in the maintenance group compared to girls, with an RRR of 0.6 (95 % CI, 0.5–0.7; *p* < .001) for dropout, 0.6 (0.5–0.8; *p* < .001) for commencement and 0.6 (0.4–0.7; *p* < .001) for nonparticipation. Participants aged 8–10 years were more likely to be in the dropout OS group and less likely to be in the commencement group rather than in the maintenance group compared to younger participants aged 6–7 years, with an RRR of 1.5 (1.3–1.9; *p* < .001) for dropout and 0.6 (0.5–0.8; *p* < .001) for commencement. Participants with parents with low education were more likely to be in the commencement and nonparticipation groups rather than the maintenance group compared to those with parents with intermediate education level, with an RRR of 1.5 (1.1–2.1; *p* = .012) for commencement and 1.5 (1.1–2.0; *p* = .010) for nonparticipation. Participants with parents with high education were less likely to be in the dropout group rather than the maintenance group compared to those with parents with intermediate education, with an RRR of 0.7 (0.5–0.9; *p* = .002). Participants with low-household income were more likely to be in the dropout, commencement and nonparticipation groups rather than the maintenance group compared to those with middle-household income, with an RRR of 1.4 (1.1–1.8; *p* = .006) for dropout, 1.5 (1.1–2.0; *p* = .008) for commencement and 2.2 (1.7–2.8; *p* < .001) for nonparticipation. Participants with high household income were less likely to be in the nonparticipation group rather than maintenance group compared to those with middle household income, with an RRR of 0.6 (0.5–0.8; *p* = .001).

#### Family-related factor

Participants not living in single-parent families were less likely to be in the commencement rather than the maintenance OS group compared to those living in single-parent families, with an RRR of 0.7 (0.5–1.0; *p* = .037).

#### Health-related factors

Participants with psychopathological problems were more likely to be in the nonparticipation group rather than the maintenance group, with an RRR of 1.6 (1.2–2.1; *p* = .001). Participants with below-average motor fitness were more likely to be in the dropout, commencement and nonparticipation groups rather than the maintenance group compared to those with average motor fitness, with an RRR of 1.4 (1.0–1.8; *p* = .026) for dropout, 1.8 (1.3–2.5; *p* < .001) for commencement and 1.7 (1.3–2.2; *p* < .001) for nonparticipation. Participants with above-average motor fitness were less likely to be in the dropout, commencement and nonparticipation groups rather than the maintenance group compared to those with average motor fitness, with an RRR of 0.7 (0.5–0.9; *p* = .002) for dropout, 0.6 (0.4–0.8; *p* = .001) for commencement and 0.4 (0.3–0.6; *p* < .001) for nonparticipation.

#### Behavioural factor

Participants with high screen-based media use were more likely to be in the commencement rather than the maintenance group compared to those with middle screen-based media use, with an RRR of 1.4 (1.1–1.9; *p* = .009).

#### Environmental factor

Participants living in rural areas were more likely to be in the nonparticipation rather than the maintenance group compared to those living in metropolitan city areas, with an RRR of 1.6 (1.0–2.6; *p* = .039).

### Subgroup analysis

Sex was an effect modifier for the associations between household income, migrant background, family form, general health status, and residential area size and OS participation. Subgroup analyses showed that these variables were associated with OS participation among girls but not among boys (see Additional file 3). Girls living in low-income households and girls living in single-parent families were more likely to be in the dropout rather than the maintenance group of OS; no associations were observed among their male counterparts (interaction term sex*household income: *p* = .045; sex*single parent: *p* = .040). Girls without a very good general health status and girls living in small- or medium-sized cities were more likely to be in the commencement rather than the maintenance group of OS; no associations were observed among their male counterparts (interaction terms sex*general health status: *p* = .015; sex*residential area size: small-sized city *p* = .036, medium-sized city *p* = .009). Girls with a migrant background were more likely to be in the nonparticipation rather than the maintenance group of OS; no associations were observed among their male counterparts (interaction term sex*migrant background: *p* = .043).

## Discussion

In this nationwide cohort study with a cluster-randomized sample of children and adolescents in Germany, the results show that 48.5 % of the children maintained their OS, 20.5 % dropped out, 12.3 % commenced and 18.7 % did not participate in OS within the 6-year observation period. In line with our observations, Vella et al. [24] also observed that the majority of the 8–9 year old Australian children in their study maintained their OS during the 2-year follow-up period until the age of 10–11 years. However, the prevalence proportion for maintenance was higher and the proportions for dropout, commencement and nonparticipation were lower in comparison to our study. Amongst other reasons, this difference might be explained by the younger average age of the Australian participants, the cultural differences (Germany versus Australia), the shorter follow-up period (2 versus 6 years) or methodological differences.

Being a girl and being in an older age group, having parents with low education, living in a low-income household and a single-parent family, having psychopathological problems, below-average motor fitness and high screen-based media use, and living in a rural area were determinants for not maintaining OS from childhood to adolescence. Five determinants were identified for OS dropout, seven for commencement and six for nonparticipation. Sex, parental education, household income, and motor fitness determined all non-maintenance OS groups (nonparticipation, dropout, and commencement), age determined dropout and commencement, family form and screen-based media use determined commencement, and psychopathological problems and residential area size determined nonparticipation in OS.

Boys showed more favourable behaviours than girls because they more often maintained their OS. Reasons for the more unfavourable behaviour among girls might be the increasing importance of their social life when they become older, which involves a shift in interests and priorities [25, 26]. Girls mature earlier than boys which might partly explain the observed gender related differences in OS participation [27]. Moreover, girls might receive less social support from their parents and peers to do sports and exercise than boys [28, 29] which might lead to higher OS dropout and nonparticipation rates. A higher OS decline and dropout among girls compared to boys was also observed in other studies [9, 30].

Compared to children who maintained their OS, children who dropped out were on average older and those who started were younger. Start of puberty is an important change in life that is accompanied by increasing demands in school, as well as change of interests, both of which can lead to declining OS participation [8, 25, 26]. This could explain why the 8–10 year old participants who were 14–16 years at KiGGS1 had a higher dropout rate than the 6–7 year old participants who were 12–13 years at KiGGS1 and who also had a higher commencement rate. The older age group reached puberty age during the follow-up, which may have led to some dropping OS, and the younger group was probably initially too young to do OS and started doing so during the follow-up. In line with this hypothesis, Rauner et al. [31] observed that from adolescence to adulthood, the dropout rate of OS continues to be higher than the commencement rate and that the discrepancy between these rates becomes even greater with increasing age. Decreasing OS participation with increasing age may be partly explained by a shift in the setting from organised sports to more self-organised fitness activities, such as going to the gym or running [32]. However, about half of the children who dropped their OS in this study (43.6 %, data not shown) indicated that they did not do any sports activity at all at KiGGS1.

Children with parents with low SES (low parental education or low household income) dropped their OS more often, commenced more often and did more often not participate; thereby showing more unfavourable behaviours compared to children with parents with a higher SES. Parents with low SES often have physically-demanding jobs and are less physically active in leisure time, whereas, parents with high SES mostly sit at work and balance their lack of PA at work with leisure-time PA [33]. PA behaviours of the parents have a direct influence on those of their children [34] and, the younger the children are, the stronger the parental control on their child’s behaviour seems to be. Therefore, parental PA and social support could be mediators between SES and OS participation, in that children of parents with low SES receive less emotional and instrumental support for OS [35, 36]. In addition, costs for equipment and member fees could be barriers for OS participation among children of parents with low SES [30, 37]. A higher OS dropout and a decline in PA among children with low SES also was observed in other studies [9, 38].

Migrant background was not a significant determinant of OS behaviour in our study, yet the RRRs point towards a higher risk for nonparticipation among children with migrant backgrounds compared to those without migrant backgrounds. Due to the small number of participants with a migrant background in our study, there was insufficient statistical power to detect any differences. Results of other studies on the association between migrant background and PA are inconsistent [39]. The comparison of study findings from different cultural settings can be flawed because migrants are heterogeneous groups. Previous German studies found that children with migrant backgrounds participated less often in OS compared to children without migrant backgrounds [40]; low levels of moderate or vigorous PA were in particular observed among girls with migrant backgrounds [41].

Children from single-parent families were more likely to commence OS, which means that they started their OS later in life than those from non-single-parent families. Children in single-parent families might receive less support from their family because of lack of time and material resources and thus start their OS when they are older and less dependent on parental support [42]. Also, Eime et al. [36] observed that girls from single-parent families were less likely to be members of sports clubs compared to girls from other families.

With the exception of psychopathological problems and motor fitness, most of the health-related variables analysed in this study were not determinants for OS participation. A possible explanation could be that a good health status is an outcome of OS participation rather than a predictor. Even though the RRRs were not statistically significant, there was a trend that a higher proportion of overweight and obese participants were categorized to be in the nonparticipation group rather than the maintenance group when compared to normal-weight participants. Furthermore, a higher proportion of those without a very good health status were categorized to be in the dropout, commencement and nonparticipation groups rather than the maintenance group when compared to those with a very good health status. Similarly, a higher proportion of those with special health care needs were categorized to be in the dropout group rather than the maintenance group compared to those with no special health care needs. Moreover, having psychopathological problems was a predictor for abstaining from OS. Children with emotional problems like anxiety could be less willing to be physically active within a group and hyperactive children who often have peer problems might also have difficulties being physically active within a group [43]. Ortlieb et al. [44] found a negative association between psychopathological problems and moderate to vigorous PA in children as well. Low motor fitness was another determinant for not maintaining OS. Children with poor motor proficiencies might have problems with learning sport-specific skills and thus possibly enjoy OS less and have poorer self-esteem. In line with our observations, Crane & Temple concluded based on their literature review that a lack of enjoyment and perception of competence are predictors for OS dropout [30] and Barnett et al. showed that motor proficiencies during childhood predicted OS participation 6 years later [45].

High screen-based media use predicted commencement of OS later in life and tended to link with OS nonparticipation. High amounts of screen-based media use during childhood may reflect a low parental awareness about the importance of PA and a low parental support for OS among their children.

Children living in rural areas had a higher risk of no participation compared to children living in metropolitan areas. Better non-organised outdoor PA possibilities in rural areas and a larger variety of and shorter distances to OS opportunities in metropolitan areas might explain this observation [37, 44]. The findings from a Canadian study are in line with our findings [46]; however, an Australian study observed the opposite, in that adolescents from rural areas had higher OS rates than those from urban areas [47].

### Strengths and limitations

KiGGS combines the advantages of high representativeness (nationwide, cluster-randomized survey with 167 sample points) and the possibility of investigating causal relationships (cohort study design). This was the first analysis that examined determinants of OS participation patterns during a 6 years transition period from childhood to adolescence using KiGGS0 and KiGGS1 data. We cannot exclude the possibility that selection bias occurred at different stages: selecting participants for KiGGS0, loss to follow-up (KiGGS1) and exclusions because of incomplete data. Response analyses conducted for KiGGS0 [48], KiGGS1 (Additional file 1) and item non-response (Additional file 2) indicate that, amongst other characteristics, participants with migrant backgrounds and low parental education were underrepresented in the study sample. In addition, we cannot exclude the possibility that participants who were less likely to participate in OS were more likely to be loss to follow-up. Thus, the presented OS dropout and nonparticipation prevalence might be underestimated in this study sample compared to the general population. Furthermore, we cannot exclude that at least some of the changes in OS participation over time occurred because of changes in the assessment mode (from proxy interview to self-report). The sample size was too small to conduct all analyses stratified by sex and, even in the total sample, statistical power was insufficient to reveal associations for some sub-group analyses (i.e., migrant background). Although motor fitness and weight status were measured objectively, most variables used were assessed based on self-reported or proxy information. We cannot exclude the possibility that reporting bias occurred (i.e., recall and social desirability bias). Misclassification bias could have occurred as well since no data were collected within the 6-year follow-up period between the measurement points (i.e., for OS participation).

## Conclusions

Despite the limitations of this study, we can conclude that more children dropped their OS than commenced and one-fifth did not participate in OS at all. There is a need for action to improve the participation rates for OS among all children and adolescents living in Germany. Health promotion efforts should focus on bringing young children into OS and preventing adolescents from stopping OS when they reach puberty age. More age and gender sensitive approaches are needed to tailor offerings which consider the preferences and interests of male and female adolescents in different life episodes. In this respect, further research should focus on the needs and preferences of adolescents as regard attractive organised sports offerings. Health policies, sports organisations and schools should cooperate to reduce barriers and increase accessibility to OS for all children and adolescents and in particular for those coming from families with low SES.

## Additional files

Additional file 1: KiGGS1 unit nonresponse analysis, difference in selected characteristics between KiGGS1 respondents and non-respondents. (DOCX 23 kb) Additional file 2: Item nonresponse analysis, difference in selected characteristics between KiGGS1 participants with and without complete data. (DOCX 24 kb) Additional file 3: Subgroup analysis, relative risk ratios for the associations between selected variables and OS participation stratified by sex. (DOCX 25 kb)

## Abbreviations

BMI: Body mass index
KiGGS: German health interview and examination survey
OS: Organised sports
PA: Physical activity
RRR: Relative risk ratio
SES: Socioeconomic status
