# Supplementary material for: Determinants of organised sports participation patterns during the transition from childhood to adolescence in Germany: results of a nationwide cohort study
Source: BMC Public Health. 2016 Sep 6;16(1):939. doi: 10.1186/s12889-016-3615-7 (PMC5012096; doi:10.1186/s12889-016-3615-7)
Supplement: Additional file 1: — KiGGS1 unit nonresponse analysis, difference in selected characteristics between KiGGS1 respondents and non-respondents. (DOCX 23 kb) [file 12889_2016_3615_MOESM1_ESM.docx]

**Additional file 1 - KiGGS1 unit nonresponse analysis**

Table 1 Difference in selected variables between KiGGS1 responder and non-responder

|  | **n** | | **Responder % (95% Cl)** | **Non-responder % (95% Cl)** | ***p*-value*** |
| --- | --- | --- | --- | --- | --- |
| **Sex** |  | |  |  |  |
| Boys | 2643 | | 51.2 (49.5-53.0) | 51.5 (48.8-54.2) | .9 |
| Girls | 2511 | | 48.8 (47.1-50.5) | 48.5 (45.9-51.2) |  |
| **Age group** |  | |  |  |  |
| 8-10 years | 3122 | | 59.4 (58.2-60.5) | 63.9 (61.5-66.3) | .004 |
| 6-7 years | 2032 | | 40.6 (39.5-41.8) | 36.1 (33.7-38.5) |  |
| **Parental education** |  | |  |  |  |
| Low | 836 | | 12.5 (11.0-14.2) | 27.5 (24.7-30.5) | < .001 |
| Intermediate | 2838 | | 55.5 (53.2-57.7) | 56.2 (52.8-59.5) |  |
| High | 1424 | | 32.0 (29.7-34.3) | 16.4 (14.3-18.7) |  |
| **Household income** | | |  |  |  |
| Low | | 1996 | 32.7 (30.6-34.9) | 58.4 (54.9-61.8) | < .001 |
| Middle | | 1733 | 37.4 (35.4-39.5) | 24.8 (22.2-27.6) |  |
| High | | 1345 | 29.9 (27.8-32.1) | 16.8 (14.5-19.5) |  |
| **Migrant background** | |  |  |  |  |
| Yes | | 756 | 10.3 (8.7-12.0) | 27.4 (23.7-31.4) | < .001 |
| No | | 4364 | 89.7 (88.0-91.3) | 72.6 (68.6-76.4) |  |
| **Overweight/obesity** | |  |  |  |  |
| Yes | | 765 | 12.9 (11.8-14.2) | 20.4 (18.5-22.4) | < .001 |
| No | | 4369 | 87.1 (85.8-88.2) | 79.6 (77.6-81.5) |  |
| **Residential area** | |  |  |  |  |
| Rural | | 1118 | 22.3 (16.4-29.6) | 19.9 (14.3-27.1) | < .001 |
| Small-sized city | | 1371 | 27.9 (21.4-35.6) | 22.9 (16.9-30.1) |  |
| Medium-sized city | | 1499 | 29.1 (22.6-36.7) | 29.0 (22.1-37.0) |  |
| Metropolitan city | | 1166 | 20.6 (15.2-27.3) | 28.2 (21.1-36.7) |  |
| **Organised sports participation** | | |  |  |  |
| Yes | 3253 | | 69.5 (67.0-71.8) | 54.6 (51.3-57.9) | < .001 |
| No | 1700 | | 30.5 (28.2-33.0) | 45.4 (42.1-48.7) |  |
| * Chi-square-test with Rao-Scott correction | | | |  |  |
