# Supplementary material for: Determinants of organised sports participation patterns during the transition from childhood to adolescence in Germany: results of a nationwide cohort study
Source: BMC Public Health. 2016 Sep 6;16(1):939. doi: 10.1186/s12889-016-3615-7 (PMC5012096; doi:10.1186/s12889-016-3615-7)
Supplement: Additional file 2: — Item nonresponse analysis, difference in selected characteristics between KiGGS1 participants with and without complete data. (DOCX 24 kb) [file 12889_2016_3615_MOESM2_ESM.docx]

**Additional file 2 - Item nonresponse analysis**

Table 1 Percentage of excluded participants from multivariate multinomial logistic regression due to missing data in at least one predictor variable stratified by selected variables

|  | **n** | **Excluded in %** | ***p*-value*** |
| --- | --- | --- | --- |
| **Total** | 380 | 11.0 | `- |
| **Sex** |  |  |  |
| Boys | 212 | 12.0 | .042 |
| Girls | 168 | 9.9 |  |
| **Age group** |  |  |  |
| 8-10 years | 207 | 10.1 | .066 |
| 6-7 years | 173 | 12.1 |  |
| **Parental education** |  |  |  |
| Low | 60 | 14.7 | .001 |
| Middle | 216 | 11.2 |  |
| High | 96 | 8.5 |  |
| **Household income** | |  |  |
| Low | 157 | 14.3 | < .001 |
| Middle | 110 | 8.5 |  |
| High | 94 | 8.9 |  |
| **Migrant background** |  |  |  |
| Yes | 102 | 31.8 | < .001 |
| No | 264 | 8.4 |  |
| **Family form** |  |  |  |
| No single-parent | 347 | 11.0 | .779 |
| Single-parent | 33 | 10.5 |  |
| **Overweight/obesity** |  |  |  |
| Yes | 44 | 9.9 | .594 |
| No | 324 | 10.8 |  |
| **General state of health** |  |  |  |
| Not very good | 218 | 11.0 | .454 |
| Very good | 150 | 10.2 |  |
| **Special health care needs** |  |  |  |
| Yes | 40 | 7.6 | .159 |
| No | 167 | 6.0 |  |
| **Psychopathological problems** | |  |  |
| Yes | 69 | 14.1 | .007 |
| No | 300 | 10.1 |  |
| **Motor fitness** |  |  |  |
| Below-average | 69 | 12.0 | .079 |
| Average | 206 | 9.3 |  |
| Above-average | 54 | 8.5 |  |
| **Screen based media use** |  |  |  |
| Low | 63 | 5.1 | < .001 |
| Middle | 92 | 7.9 |  |
| High | 89 | 9.5 |  |
| **Residential area** |  |  |  |
| Rural | 65 | 8.5 | .067 |
| Small-sized city | 110 | 11.1 |  |
| Medium-sized city | 126 | 12.4 |  |
| Metropolitan city | 79 | 11.2 |  |
| **Organised sports participation** | |  |  |
| Maintenance | 165 | 9.6 | .002 |
| Dropout | 73 | 10.4 |  |
| Commencement | 50 | 11.4 |  |
| Nonparticipation | 92 | 15.2 |  |

*Chi-square-test with Rao-Scott correction
