# Supplementary material for: Determinants of organised sports participation patterns during the transition from childhood to adolescence in Germany: results of a nationwide cohort study
Source: BMC Public Health. 2016 Sep 6;16(1):939. doi: 10.1186/s12889-016-3615-7 (PMC5012096; doi:10.1186/s12889-016-3615-7)
Supplement: Additional file 3: — Subgroup analysis, relative risk ratios for the associations between selected variables and OS participation stratified by sex. (DOCX 25 kb) [file 12889_2016_3615_MOESM3_ESM.docx]

**Additional file 3 – Subgroup analysis**

Table 1 Results of the multinomial logistic regression for the association between household income and dropout as well as family form and dropout stratified by sex

|  | | **No. in sample** | **Dropout** |
| --- | --- | --- | --- |
| **Stratified analysis by sex** | |  | RRR (95% Cl) |
| **a** | **Boys** |  |  |
|  | Low income household | 110 | 1.1 (0.8-1.5) |
|  | Middle income household | 128 | 1.0 |
|  | High income household | 83 | 0.9 (0.6-1.3) |
|  | **Girls** |  |  |
|  | Low income household | 127 | 1.8 (1.3-2.6)* |
|  | Middle income household | 126 | 1.0 |
|  | High income household | 127 | 1.3 (0.9-1.8) |
| **b** | **Boys** |  |  |
|  | No single-parent | 296 | 1.3 (0.7-2.2) |
|  | Single-parent | 25 | 1.0 |
|  | **Girls** |  |  |
|  | No single-parent | 336 | 0.6 (0.4-1.0)* |
|  | Single-parent | 44 | 1.0 |
| **p*-value < .05; models adjusted for age, parental education, household income (b), migrant background, family form (a), weight status, general state of health, special health care needs, psychopathological problems, motor fitness, screen-based media use, and residential area; RRR = Relative risk ratio | | | |

Table 2 Results of the multinomial logistic regression for the association between general state of health and commencement as well as residential area and commencement stratified by sex

|  |  | **No. in sample** | **Commencement** |
| --- | --- | --- | --- |
| **Stratified analysis by sex** | |  | RRR (95% Cl) |
| **a** | **Boys** |  |  |
|  | Not very good state of health | 88 | 0.9 (0.6-1.2) |
|  | Very good state of health | 115 | 1.0 |
|  | **Girls** |  |  |
|  | Not very good state of health | 86 | 1.6 (1.1-2.2)* |
|  | Very good state of health | 148 | 1.0 |
| **b** | **Boys** |  |  |
|  | Rural | 55 | 1.8 (0.9-3.3) |
|  | Small-sized city | 49 | 1.2 (0.7-2.1) |
|  | Medium-sized city | 62 | 1.4 (0.8-2.4) |
|  | Metropolitan city | 37 | 1.0 |
|  | **Girls** |  |  |
|  | Rural | 69 | 1.3 (0.8-2.2) |
|  | Small-sized city | 54 | 0.6 (0.4-1.0)* |
|  | Medium-sized city | 57 | 0.6 (0.4-1.0)* |
|  | Metropolitan city | 54 | 1.0 |
| **p*-value < .05; models adjusted for age, parental education, household income, migrant background, family form, weight status, general state of health (b), special health care needs, psychopathological problems, motor fitness, screen-based media use, and residential area (a); RRR = Relative risk ratio | | | |

Table 3 Results of the multinomial logistic regression for the association between migrant background and nonparticipation stratified by sex

|  | **No. in sample** | **Abstinence** |
| --- | --- | --- |
| **Stratified analysis by sex** |  | RRR (95% Cl) |
| **Boys** |  |  |
| Migrant background | 33 | 0.9 (0.5-1.7) |
| No migrant background | 235 | 1.0 |
| **Girls** |  |  |
| Migrant background | 52 | 2.1 (1.2-3.8)* |
| No migrant background | 285 | 1.0 |
| **p*-value < .05; models adjusted for age, parental education, household income, family form, weight status, general state of health, special health care needs, psychopathological problems, motor fitness, screen-based media use, and residential area; RRR = Relative risk ratio | | |
